# Influencing factors of SARS-Cov2 spread in Africa

**DOI:** 10.7189/jogh.10.020331

**Published:** 2020-12

**Authors:** Brahim Admou, Raja Hazime, Imane Brahim, Ahmed Rhassane El Adib

**Affiliations:** 1 Center of Clinical Research, Laboratory of Immunology, University Hospital Mohammed VI, Marrakech, Morocco; 2 Bioscience Research Laboratory, Faculty of Medicine, Cadi Ayyad University, Marrakech, Morocco; 3 Department of Anesthesiology, Intensive Care and Emergency Medicine, University Hospital Mohammed VI, Marrakech, Morocco

More than 5 months after the COVID-19 outbreak, high-level endemic transmissions occurred in different regions of the globe with shocking tournaments even in developed countries [1].

After the warnings launched by the World Health Organization (WHO) the spotlights are currently turned towards African countries. What might happen to Africa, where most countries have weak health care systems, including inadequate surveillance and laboratory capacity, scarcity of public health human resources, and limited financial means [2]. Nevertheless, to better manage this multidimensional crisis, the challenge is not only about the availability of health infrastructures, but also how to considerate other factors that may modify the course of the disease by either accelerating or rather limiting the spread of the virus, such as geographical, socio-economical, and even political factors.

## GEODEMOGRAPHIC AND CLIMATIC FACTORS

By the date of May 26th, and irrespective of possible non declared cases presumably because of possible limited testing and reporting capabilities, all African countries are nowadays facing COVID-19 with a total of 124 482 confirmed cases, with 69691 active cases, distributed as follow: North (37600 cases), Western (31300 cases), Southern (27900 cases), Central (13900 cases), and Eastern Africa (13800 cases) [3].

Due to their geographic position, certain regions would be more affected by the start of the COVID-19 outbreak since they are at the greatest level of international contact like trade, tourism, diplomatic travel, study or business, then can be among the hardest hit of this pandemic [2]. This fact may explain the significant COVID-19 cases in North African, South African and Western countries as well, added to the geographic distribution of countries, which easily allows predicting more cases in high density zones. Moreover, urban areas, especially the administrative and/or economical capitals or megacities are often remarkably densely populated, which creates conditions where virus can spread rapidly and may remain undetected, whereas rural and Saharan areas should take a great advantage of geographic distance and lack of promiscuity to be at very low risk of contamination.

Furthermore, the particular large African youth population may lead to more infections but most of them will be asymptomatic or with minor symptoms, and will probably go undetected, with a risk of infecting more people than would symptomatic individuals [4].

On the other hand, it was demonstrated that climatic conditions limit the geographical and seasonal distribution of emergent infectious diseases, and weather affects the timing and intensity of their outbreaks. In fact, the climate-specific differences between regions, the effect of UV light on the survival of the virus on surfaces, or the higher temperatures my significantly impact the current SARS-CoV2 spread [5]. However, other authors affirm that SARS-CoV2 can be transmitted in all areas, including those with hot and humid weather, and there is no evidence supporting the hypothesis that the virus will spread more slowly in countries with warmer climates.

## COVID-19 AND HEALTH SYSTEM SETTINGS

All Africa, especially low and middle income countries (LMIC) are preparing for the potential impact of the pandemic, with the risk of overwhelming their already fragile health systems [6], but many of them are suffering from scarce critical care resources, limitations in the availability of basics, as well as health care provider shortages related to COVID-19 services, such as testing and treatment to be provided freely to all citizens [7]. In several other countries, Intensive Care Unit (ICU) beds and staff trained in critical care are mostly limited to tertiary hospitals. Therefore, mortality associated with COVID-19 is likely to exceed the average death rate of the pandemic, mostly vulnerable and immunocompromised populations who are at greater risk of developing severe and critical disease [8].

Other questions are also raised about whether the virus genotypes and mutations contribute to host tropism and rapid global spread. Thus, alongside the clinical management of patients, health systems need to closely monitor the genome of SARS-CoV2 in each country, in order to determine its virulence and possible future mutant strains, with the potential impact of this on the propagation of the virus.

## COVID-19 AND LEARNT LESSONS FROM THE HISTORY

The continent already faces existing endemic diseases, such as HIV, tuberculosis, and malaria; remerging and emerging infectious pathogens, such as Ebola virus disease, and others; and increasing incidence of non-communicable diseases [2,6].

Interestingly, HIV, Ebola virus, and SARS-CoV2 are all of zoonotic origin. Most of them resulted on cross-species transfer from animals in Eastern and Central Africa to humans.

This fact may show the potential impact of COVID-19 on already fragile health systems, particularly in countries suffering from a lack of sufficiently trained personnel, and limited resources, added to highly porous international borders [7].

Conversely, these experiences may prove to be an advantage for generations of already sensitized governments and communities to undertake rapid, proactive and adapted measures during the current pandemic.

On the other hand, the differences in immunological background of the population, pre-exposure with coronaviruses or other infections may confer a certain degree of immunization against SARS-CoV2 in many areas [5]. For example, besides its specific effect against tuberculosis, the BCG vaccine has beneficial nonspecific effects on the immune system that protect against a wide range of other infections. This has led to the suggestion that vaccination with BCG might have a role in protecting health care workers and other vulnerable individuals against severe COVID-19. It might therefore reduce viremia after SARS-CoV2 exposure, with less severe cases and more rapid recovery [9].

## SOCIO-CULTURAL FACTORS

Africa is known for its well-merited reputation of community, and many daily activities turn around social interactions between people, including mosque, church, market shopping or even enjoying time in a cafe. Therefore, any strategies adopted need to recognize local socio-cultural rituals and challenges. Social distancing will be difficult to implement and sustain, particularly in crowded neighborhoods where many generations often cohabit, or for individuals who are food-seeking or require daily pay [1,10]. Despite these constraints, in almost African countries, all schools, mosques and churches have already been closed to promote social distancing; this would certainly have a beneficial impact on limiting the spread of the virus.

In rural areas, the traditional leader or the tribal chief who is generally supported by the local authorities represents a kind of leadership who has a considerable authority; his ability to deliver various responsibilities in accordance with the local establishment, his legitimacy and influence remain prevalent and may allow him to participate effectively in regulating the social behavior at the era of COVID-19 pandemic.

Additionally, citizens' support for government efforts to mitigate the impact of the pandemic is closely linked to their trust in the government in terms of transparency, which engenders a feeling of solidarity, and reinforces the conviction among citizens that the mitigating measures are applied fairly.

## COVID-19 AND LEADER’S RESPONSIBILITIES

Besides the huge efforts to upgrade the health system for a better management of COVID-19 patients, African governments need to scale up the containment and mitigation strategies related to quarantine, isolation and social distancing, with restrictions or absolute bans on social gatherings [6,10].

**Figure:**
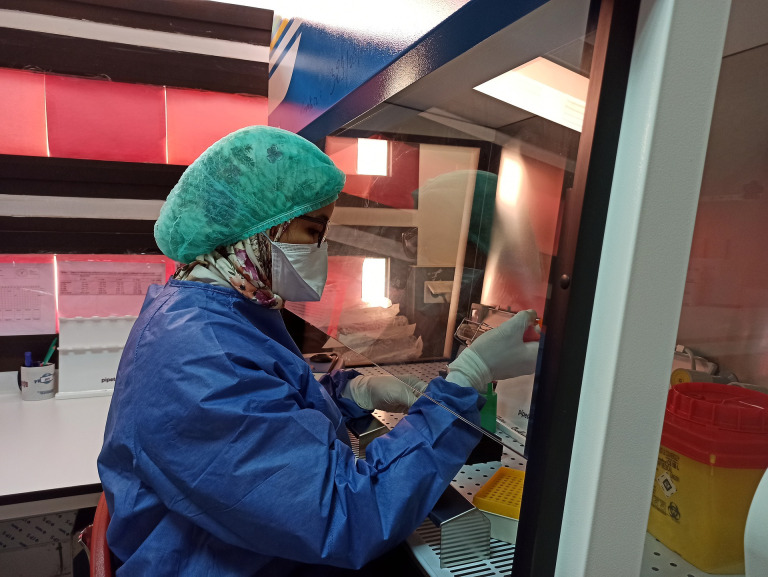
Photo: Laboratory technician performing immunological analysis related to COVID-19 at the University Hospital of Marrakech, Morocco (from Brahim Admou’s collection, used with permission).

Fortunately, as the pandemic makes its first incursions, several countries have established vigorous measures in collaboration with public health departments to isolate people having close contact with infected or suspected individuals. Other measures taken have been shown to be very effective in limiting the spread of the pandemic despite their major economic damage like closing borders, shuttering markets, suspending both international and internal flights [10].

In parallel, governments should focus on laboratory diagnosis and surveillance, including screening at points of entry and cross-border activities; infection prevention and control in health care facilities [5].

In a frame of an international collaboration, LMIC should be fully supported technically and financially by WHO and other governments, including donations of coronavirus test kits, personal protection tools, and other life-support equipments or, at a minimum, ensuring that African countries are not priced out of the market for these commodities [10].

## THE ROLE OF MEDIA

The role of media in fighting the pandemic is crucial. In fact, both positive and negative messaging can considerably influence the attitude of the population, thus they have an obligation to contribute both in preparedness and controlling the outbreak in collaboration or even in synergy with government, to provide consistent, simple, clear and motivational messages. Furthermore, health officials need to use diverse communication channels to disseminate information about the progress of the outbreak, and the interventions being implemented in a transparent and timely manner [7].

To achieve the greatest impact, consistent and credible messaging that is coordinated among key traditional and religious leaders and political actors is also critical [7].

Countries need also to mobilize virtual learning networks to disseminate information to health and community workers and to the public in culturally appropriate messages, such as daily briefings. Indeed, simple health messaging will greatly improve the confidence of the public in the government efforts to shorten the spread of this virus [10].

## CONCLUSION

Among the factors linked to the spread of SARS-CoV2, some are natural and uncontrollable (geography, climate), but many even varied and at times overlapping are within reach of African governments and populations as well.

To avoid the worst of the pandemic, strong political leadership and coordinated efforts are needed to ensure a good epidemic preparedness, including: successful containment; early detection and testing freely provided and available nationwide to all citizens; adapted health system infrastructure; active surveillance; isolation and case management; contact tracing and prevention of onward spread of SARS-CoV2.

The role of media in fighting the pandemic and limiting the virus dissemination is crucial with coordinated efforts with all civil society actors.

Finally, COVID-19 reminds us that Africa will not forgive us if we fail to accomplish our duties during this threatening pandemic.
